# A multifaceted strategy using mobile technology to assist rural primary healthcare doctors and frontline health workers in cardiovascular disease risk management: protocol for the SMARTHealth India cluster randomised controlled trial

**DOI:** 10.1186/1748-5908-8-137

**Published:** 2013-11-25

**Authors:** Devarsetty Praveen, Anushka Patel, Stephen McMahon, Dorairaj Prabhakaran, Gari D Clifford, Pallab K Maulik, Rohina Joshi, Stephen Jan, Stephane Heritier, David Peiris

**Affiliations:** 1 The George Institute for Global Health, Hyderabad, India; 2 The University of Sydney, Sydney, New South Wales, Australia; 3 The George Institute for Global Health, The University of Sydney, Sydney, New South Wales, Australia; 4 Centre for Chronic Disease Control, New Delhi, India; 5 Institute of Biomedical Engineering, Department of Engineering Science, University of Oxford, Oxford, UK; 6 The George Institute for Global Health, Oxford University, Oxford, UK

**Keywords:** Blood pressure, Capacity strengthening, Clinical decision support system, Healthcare workers, Physicians, India, Implementation

## Abstract

**Background:**

Blood Pressure related disease affected 118 million people in India in the year 2000; this figure will double by 2025. Around one in four adults in rural India have hypertension, and of those, only a minority are accessing appropriate care. Health systems in India face substantial challenges to meet these gaps in care, and innovative solutions are needed.

**Methods:**

We hypothesise that a multifaceted intervention involving capacity strengthening of primary healthcare doctors and non-physician healthcare workers through use of a mobile device-based clinical decision support system will result in improved blood pressure control for individuals at high risk of a cardiovascular disease event when compared with usual healthcare. This intervention will be implemented as a stepped wedge, cluster randomised controlled trial in 18 primary health centres and 54 villages in rural Andhra Pradesh involving adults aged ≥40 years at high cardiovascular disease event risk (approximately 15,000 people). Cardiovascular disease event risk will be calculated based on World Health Organisation/International Society of Hypertension’s region-specific risk charts. Cluster randomisation will occur at the level of the primary health centres. Outcome analyses will be conducted blinded to intervention allocation.

**Expected outcomes:**

The primary study outcome is the difference in the proportion of people meeting guideline-recommended blood pressure targets in the intervention period vs. the control period. Secondary outcomes include mean reduction in blood pressure levels; change in other cardiovascular disease risk factors, including body mass index, current smoking, reported healthy eating habits, and reported physical activity levels; self-reported use of blood pressure and other cardiovascular medicines; quality of life (using the EQ-5D); and cardiovascular disease events (using hospitalisation data). Trial outcomes will be accompanied by detailed process and economic evaluations.

**Significance:**

The findings are likely to inform policy on a scalable strategy to overcome entrenched inequities in access to effective healthcare for under-served populations in low and middle income country settings.

**Trial registration:**

Clinical Trial Registry India CTRI/2013/06/003753.

## Background

### Cardiovascular disease (CVD) burden in India

In India, the number of years of life lost due to CVD deaths among populations 35 to 64 years of age will increase from 9.2 million in 2000 to 17.9 million in 2030 – more life years lost than is projected for China, Russia, and the USA combined [1]. Elevated blood pressure (BP) is a major contributor to the increasing burden of CVD in India, estimated to be responsible for a million deaths annually [2]. India had an estimated 118 million people diagnosed with hypertension in 2000 with projections indicating a near doubling to 213 million by 2025 [3]. Around 16% of ischaemic heart disease, 21% of peripheral vascular disease, 24% of acute myocardial infarctions, and 29% of strokes in India are considered attributable to hypertension [4]. This disease burden is not confined to urban India. In rural areas, where 70% of the country’s population resides, high levels of hypertension and other CVD risk factors exist, with CVD already the leading cause of adult death in many such communities [5,6].

### Evidence-practice gaps in CVD prevention

Use of BP lowering treatments in rural India is limited, even where mandated low cost drugs are available in government formularies. Earlier studies done in rural communities in India have shown that few people with hypertension and/or CVD are appropriately managed [7,8]. In rural Andhra Pradesh, 27.0% of adults aged ≥30 years had hypertension with around one-half previously unidentified [6,8-10]. In these communities, 7.7% of adults had established CVD, and a further 8.1% had a 10-year CVD risk ≥ 30%. For those with CVD, 25% had non-optimal BP levels (systolic BP >140 mmHg). For those at high risk without known CVD, 95% had non-optimal BP levels. These rates were similarly experienced by men and women. Overall, only 39% of all high risk individuals with or without CVD have adequate BP control, indicating large evidence-practice gaps. In the context of limited resources, prioritising high-risk patients for BP lowering treatment is likely to be a highly cost-efficient approach and is consistent with Indian guideline recommendations.

### Workforce challenges

India’s health system faces great challenges in tackling a rapidly escalating burden of CVD. Key issues include lack of healthcare facilities, limited access to healthcare providers, and high out-of-pocket costs for consumers [11]. It is therefore imperative that innovative solutions are developed to address these issues. India’s three tier healthcare system provides nurse/midwife level primary healthcare at the sub-centre (population approximately 3,000 to 5,000), doctor-level care at the Primary Health Centre (PHC) (population approximately 20,000 to 30,000), and specialised care at the Community Healthcare Centres (population approximately 80,000 to 120,000). The PHC, which is usually led by one doctor, is expected to provide comprehensive primary healthcare for up to 30,000 residents. This leads to considerable strain on PHC resources potentially resulting in inadequate quality of care.

In this context, there is an urgent need for innovations in healthcare delivery. One promising strategy involves ‘task shifting,’ where front-line, non-physician health workers are delegated some of the tasks traditionally performed by physicians. In the setting of HIV/AIDS care, task-shifting has been shown to improve health outcomes and processes of care [12]. In India, there is some evidence that task shifting CVD risk assessment to non-physician health workers via a simple algorithm can increase the detection of CVD [13]. An example of front-line health workers in India is Accredited Social Health Activists (ASHAs), who are local female residents from the village. ASHAs have an average of ten years of formal education and are selected for this role by the village Panchayat (a village based self-governance system) and receive performance-based remuneration under India’s National Rural Health Mission program. On average, they work for two to three hours each day. Until now they have primarily been deployed to provide maternal and child health services via household visits. This suggests that this workforce can be trained to effectively identify people at high risk and refer them appropriately for medical care. In this study, the ASHA model is used as a platform for the integrated delivery of cardiovascular disease risk factor screening.

### Clinical decision support systems (CDSS)

In five systematic reviews on the effectiveness of CDSS, approximately two-thirds of controlled studies have demonstrated improvement in healthcare performance [14-19]. The vast majority of high quality trials, however, have been conducted in high income countries and have targeted physicians and other healthcare workers with high levels of training. The external validity of CDSS in Low and Middle Income Country (LMIC) settings is unclear [20]. One promising strategy to increase the uptake of CDSS in resource limited settings is through the use of mobile devices (mobile phones, smartphones and tablets). Given their increasing ubiquity, these devices represent one of the few hardware products available with the potential to transform the delivery of essential healthcare on a large scale. Research evidence to demonstrate this, however, is still relatively nascent. Despite the promise of an ‘m-health’ revolution in LMICs, a recent comprehensive review concluded that the current evidence for their effectiveness is fragmented and focused on intermediary outcomes such as cost savings and improved data quality [21].

### SMARTHealth – mobile decision support development

To help address these research gaps, we developed a novel comprehensive CDSS to facilitate Systematic Appraisal Referral and Treatment of CVD risk in rural India (SMARTHealth India). It has been designed for use by ASHAs and PHC doctors. A single screening and management algorithm was developed based on Indian and international guidelines. World Health Organisation/International Society of Hypertension (WHO/ISHs) region-specific risk charts are used to calculate a person’s ten-year absolute CVD event risk. For management recommendations, the Indian National Program for Prevention and Control of Cancer, Diabetes Cardiovascular and Stroke (NPCDCS) guidelines were programmed [22].

The algorithm was programmed into an application on a 7-inch tablet using Android 4.1 operating system. Both English and local language versions (Telugu) were developed. It was validated using a three-stage process based on our previous work [23]. Stage 1 was an iterative process where output from the algorithm was tested using de-identified data from patients in a previous rural Indian study and programming modifications were made. Stage 2 involved independently programming the algorithm into a statistical software package (SPSS v21.0, Armonk, NY). Primary de-identified healthcare data from 1,000 patients were imported into the CDSS and the SPSS program. For all output variables, the correlation (continuous variables) and agreement (categorical variables) between the two programs were assessed. A few minor coding errors in the CDSS were identified, rectified and re-tested until perfect correlation/agreement was achieved. Stage 3 involved user acceptance testing and field testing with doctors and ASHAs in 11 villages and 3 PHCs. Detailed analyses of the development and pilot phase will be published separately. Following the field testing, refinements were made to the system to make it system-ready for trial implementation.

## Research methods

### Study objectives

The SMARTHealth India study will test whether a CDSS will assist non-physician health workers and doctors in making evidence based management decisions to lower their patients’ CVD risks. The hypothesis is that a multifaceted intervention involving capacity strengthening of primary healthcare doctors and non-physician healthcare workers through use of a mobile device-based clinical decision support system will result in improved BP control for individuals at high CVD event risk when compared with usual healthcare.

### Study design

The intervention will be evaluated using a stepped-wedge cluster randomised, controlled trial (cRCT) of two years duration.

### Study population

A total of 18 PHCs (3 villages per PHC) in West Godavari District, Andhra Pradesh, will participate (see Statistical considerations below). Patients will be eligible to participate if they are residents of the study sites, are ≥40 years of age, are classified at high CVD risk and are indicated for BP lowering treatment based on WHO and NPCDCS guidelines.

High CVD risk is defined as the presence of any of the following:

1. Past history of CVD.

2. Estimated 10-year CVD risk ≥30%.

3. Estimated10-year CVD risk of 20% to 29% and a Systolic BP >140 mmHg.

Risk will be calculated using WHO/ISH algorithms for India.

### Randomisation

Cluster randomisation will occur at the level of the PHC. A total of 18 PHCs from West Godavari District in Andhra Pradesh will be selected. To be eligible, all PHCs must have at least one doctor regularly providing services, and all doctors must be willing to participate in the study. From the region serviced by each PHC, three villages will be randomly selected (54 villages in total). Six PHCs (18 villages) will be randomised to the intervention over 3 time intervals or steps (Table 1). Permuted block randomisation will be centrally performed at the George Institute in Hyderabad using a web-based form stratified by PHC size. Outcome analyses will be conducted blinded to intervention allocation.

**Table 1:** Stepped-wedge randomisation of the participating villages to the intervention group in three time intervals or steps

	**Time interval**			
Number	Month 0–6	Month 7–12	Month 13–18	Month 19–24
6 PHCs (18 villages)	CONTROL	**INTERVENTION**	**INTERVENTION**	**INTERVENTION**
6 PHCs (18 villages)	CONTROL	CONTROL	**INTERVENTION**	**INTERVENTION**
6 PHCs (18 villages)	CONTROL	CONTROL	CONTROL	**INTERVENTION**

A stepped-wedge design will ensure that every participating PHC and village receives the intervention (for at least 6 months and an average of 12 months), while still allowing an unbiased evaluation of the effectiveness of the intervention compared to usual care.

### Intervention components

**Figure 1:**
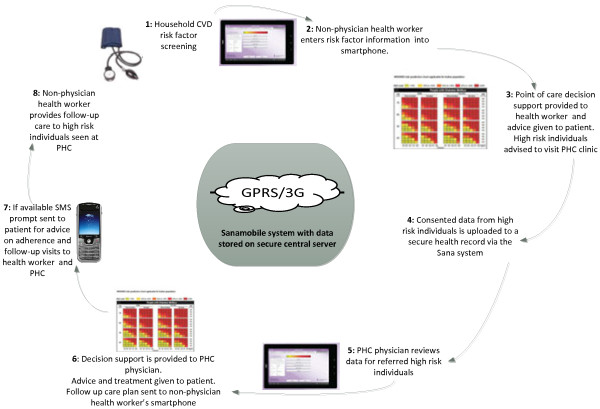
Workflow and the key elements of the intervention package.

SMARTHealth allows health workers to collect consented patient information for screening and healthcare purposes and process this information based on the algorithm described above. The application then uploads this information for a doctor to review using OpenMRS – a secure, community-developed, open source, electronic medical record system platform. ASHAs can make electronic referrals to the PHC doctor, and doctors can notify the health worker via his/her tablet of the diagnosis and management plan. Figure 1 outlines the workflow and the key elements of the intervention package. The intervention comprises the following:

1. Equipment for ASHAs and PHC doctors to assess and manage CVD risk using the CDSS application in a 7-inch Smart Tablet. A backpack-sized kit, containing Smart Tablet, BP monitor, glucometer and other management resources will be provided.

2. The application will also support ASHAs to promote lifestyle advice for the determinants of high blood pressure and CVD, in particular physical activity, healthy diet and avoidance of tobacco and alcohol. There will be no out-of-pocket costs for participants accessing this service.

3. Training and resource support to ASHAs and PHC doctors. ASHAs will be provided with training in taking a patient history and measurement of BP and other CVD risk factors via the CDSS, provision of lifestyle advice to lower CVD risk; referral of high risk individuals and follow-up once seen by the doctor. Doctors will similarly be trained in use of the CDSS and additionally provided with training on pharmacological management.

4. A shared electronic record functionality using Open MRS and Sana to capture patient information via Smart Tablet and securely send data to a centralised server.

5. The doctors will use the electronic data transmitted by the ASHAs and interpret the decision support output for management of BP and other CVD risk factors. The decision support is based on current Indian national guidelines and will provide recommendations for BP lowering, lipid lowering, or anti‒platelet medications. The doctors will be advised to prescribe medications from these drug classes that are available on the essential medicine list in primary healthcare facilities to minimise out of pocket expenses.

6. A prompt system for follow-up of high risk individuals by ASHAs and doctors.

7. Both ASHAs and physicians have a permitted private practice allocation and will be remunerated accordingly. This is actively encouraged by district medical health officers and will ensure study participation will not divert the ASHA or the PHC doctor from their usual duties addressing other health priorities for the population.

### Control arm

During control periods, access to healthcare will continue as per usual practice without the ASHAs and PHC doctors having access to the CDSS, associated tools, and the training and support package.

### Data collection

**Figure 2:**
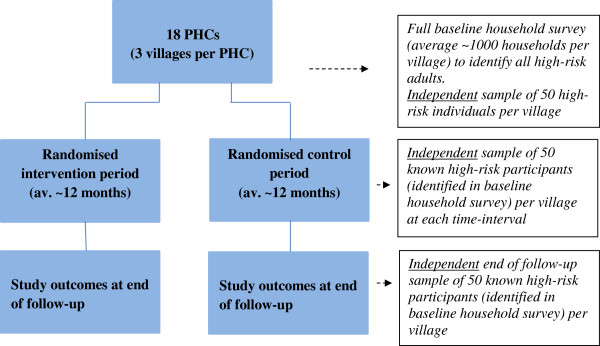
SMARTHealth study schema.

The study schema is outlined in Figure 2. Independent data collection will be conducted by trained household surveyors based on previous well-established and acceptable methods [6,8-10,13]. Collection will occur on five occasions for each village – at baseline, at each interim time-interval (*i.e*., each ‘step,’ see Table 1), and at the end of follow-up. This allows unbiased evaluation of effectiveness through comparison of ‘control periods’ (for each village, the period between baseline and pre-intervention) and ‘intervention periods’ (for each village, the period between pre-intervention and end of follow-up).

At baseline, a complete household survey (average of approximately 1,000 households per village) will be performed in each village. Trained field researchers will conduct interviews and make physical measurements using well-established methods [6]. In each household, every consenting adult aged ≥40 years of age will be assessed, and those at high risk of CVD will be identified, resulting in a census of all such individuals. Any individuals with extreme elevations of risk factors will be referred immediately for treatment. At each subsequent time point, data will be collected from a random independent sample of 15% of people identified at the baseline census as being at high risk (average of approximately 50 people per village). These data are for evaluation purposes only, and the ASHA and PHC doctors will not have access to this information.

### Primary outcomes

1. Difference in proportion of high risk individuals (with or without CVD) who are achieving optimal BP levels (systolic BP <140 mmHg) between the intervention and control periods.

### Secondary outcomes

1. Mean reduction in BP levels.

2. Change in other CVD risk factors, including body mass index; current smoking; reported healthy eating habits; and reported physical activity levels.

3. Self-reported use of BP and other cardiovascular medicines.

4. Quality of life (using the EQ-5D).

5. CVD events (using hospitalisation data).

### Statistical considerations

A total of 18 PHC clusters (with 3 villages per cluster) progressively randomised by a third to the intervention (Table 1) will provide >90% power (2α = 0.05) to detect an absolute difference of 6% in the proportion of people with optimal BP levels. This translates to an increase in the proportion achieving optimal BP levels from 39% (based on our previous data) to 45% and a mean systolic BP difference of around 3 mmHg. These calculations maximise the study power afforded by a stepped-wedge design and assume an intraclass correlation coefficient (ICC) of 0.03 (more conservative than the ICC of 0.01 previously observed in this population) and 5 time-points for data collection. The mean village population is approximately 4,000, with one-third aged ≥40 years. Based on previous data from the region, approximately 23% of adults aged ≥40 years (approximately 300 individuals per village) are likely to be classified as being at high risk, with around 35% of these having established CVD [9]. Considering a conservative participation rate at each survey, a PHC cluster size of 150 patients per time point (approximately 50 per village) is anticipated on the basis of the above calculation. Methods based on mixed effects models as described by Hussey and Hughes will be used to analyse intervention effectiveness on primary and secondary outcomes, accounting for outcome variable type, potential time effects, clustering and stepped wedge design [24].

### Economic and process evaluation

The economic evaluation will have a trial-based component and a modelled evaluation of long-term costs and outcomes. The intervention cost will be based on salaries, training, equipment and other costs incurred with implementation of the intervention. Trial-based data, however, cannot capture costs and outcomes beyond the trial. A decision-analytic model will enable long-term cardiovascular morbidity, quality of life and survival to be simulated. Cost-effectiveness will be calculated in terms of incremental cost per quality adjusted life years gained. This will better inform policy makers as to the resource consequences of rolling out this program to scale.

A detailed awareness of local contextual factors will be critical to understanding the impact of the intervention and any barriers to its implementation. The process evaluation will be informed by behaviour change theory [25], assessing how well the new system of service provision fits within the usual processes of current service provision in the villages and PHCs centres. A mixed methods approach to investigate why the intervention strategy may or may not have been effective and which intervention components were most influential will be used. Three data sources will be used: de-identified usage data from the tablet based system; patient and provider surveys; and semi-structured interviews with a purposive sample of participants and care providers toward the end of study.

### Ethical considerations

The study is approved by the Centre for Chronic Disease Control Institutional Ethics Committee (IEC), New Delhi and the University of Sydney Human Research Ethics Committee (HREC), NSW, Sydney. It is also registered in the Clinical Trials Registry of India. The study has also been discussed with each village Panchayat. Informed consent will be obtained from all participants contributing data before the randomisation process. All data collection and reporting will be compliant with national privacy law, and no report will allow an individual participant to be identified. Data will be securely stored at the George Institute Hyderabad. All study records and documents will be stored for a minimum of seven years from the end of the study or for a period as required by any individual HREC.

### Trial status

The study will be initiated with a household baseline survey in all 54 villages by the fourth quarter of 2013. Randomisation of PHCs and follow-up village surveys of high risk individuals will continue until the first quarter of 2016, followed by analysis and dissemination of the findings in middle of 2016.

## Discussion

SMARTHealth India focuses on a ‘real life’ implementation of a complex intervention. It represents a case study into ‘Integrated Innovation’ [26], incorporating a science/technology component (Smart Tablet, CDSS, and cutting edge trial design), a social component (innovative workforce strategies), and a business component (integration with existing health system infrastructure). Despite great promise for m-health interventions to improve access to effective healthcare, there remains considerable uncertainty about how this can be successfully achieved. These uncertainties pose substantial dilemmas for health system planners, particularly in LMICs. This project will rigorously explore the challenges of implementing well-established evidence into practice.

### Strengths and limitations

The strengths of the study are that it explores the challenges of implementing a complex intervention, taking into consideration policy, healthcare providers, and consumer perspectives. The evidence generated thus has substantial potential to inform decision-making for system planners on a scalable solution to increasing access to high quality primary healthcare for common chronic conditions.

The main limitation is that it is conducted in one rural region of one country, and the findings may not be generalizable to elsewhere. Integrating the intervention with the existing primary healthcare system, which is broadly similar in all parts of India, will help to mitigate this and enhance the relevance of the results across India and other regions with similar health system structures. Another potential limitation is that by using a stepped-wedge design, secular changes in the region during the study period may bias the outcomes. However, considering the study duration, time adjustment in the analysis, and recommendations by key stakeholders that access to the intervention be provided to all centres, this design provides, on balance, the most acceptable method to test the study hypothesis in a rigorous but pragmatic manner.

### Significance

The findings from this study are likely to advance locally relevant knowledge on scaling up a strategy to overcome entrenched inequities in access to effective healthcare for under-served populations. Such approaches, if found to be effective and cost-effective and combined with effective population-based strategies, have the potential to positively impact the healthcare of millions of Indians on a daily basis and will have wider applicability for other LMICs.

## Abbreviations

BP: Blood pressure; CVD: Cardiovascular diseases; PHC: Primary health centre; ASHA: Accredited social health activist; CDSS: Clinical decision support system; LMIC: Low and middle income countries; WHO/ISH: World health organisation/international society of hypertension; NPCDCS: National programme for cancer, diabetes, cardiovascular diseases and Stroke; cRCT: Cluster randomised controlled trial; IEC: Institutional ethics committee; HREC: Human research ethics committee.

## Competing interests

The authors declare that they have no competing interests.

## Authors’ contributions

DPe and AP conceptualised the study and detailed the main content of the paper. DPrav leads the study in India, is responsible for all implementation aspects of the study, and prepared the initial manuscript draft. GC contributed to the technical aspects of the intervention development. SJ contributed to the economic evaluation design. SH contributed to the statistical aspects of the study. SM, DPrab, SH, PM, RJ contributed to the overall study design. All authors contributed to drafting the manuscript, and all have read and approved the final manuscript.
